# Patients and treatments in a neuropalliative outpatient clinic: an analysis of clinical routine data from five years of care

**DOI:** 10.3389/fneur.2025.1616153

**Published:** 2025-09-05

**Authors:** Teresa Grimm, Fabian Otto-Sobotka, Dorothee Steinker, Oliver Summ, Antje Timmer, Martin Groß

**Affiliations:** ^1^Department of Neurological Intensive Care and Rehabilitation, Evangelisches Krankenhaus Oldenburg, Oldenburg, Germany; ^2^Faculty VI Medicine and Health Sciences, Carl von Ossietzky Universität Oldenburg, Oldenburg, Germany; ^3^Department of Epidemiology and Biometry, Faculty VI Medicine and Health Sciences, Carl von Ossietzky Universität Oldenburg, Oldenburg, Germany; ^4^Department of Neurology, MEDIAN Klinik Bad Tennstedt, Bad Tennstedt, Germany

**Keywords:** neuropalliative care, palliative treatment, neuropalliative outpatient clinic, life-threatening diseases, neurodegenerative diseases, long-term neurological conditions

## Abstract

**Introduction:**

The increasing prevalence of life-threatening neurological diseases raises the need for neuropalliative care. Setting up neurological palliative outpatient clinics is one way of addressing this need. This study aims to describe the patient clientele of a neurological palliative outpatient clinic and the spectrum of necessary treatments and interventions.

**Methods:**

In this longitudinal analysis, clinical routine data from a single centre were collected retrospectively from adult patients. The patient characteristics related to disease and treatment were evaluated descriptively. Factors influencing the need for ventilation were modelled in a logistic regression. The required treatment effort was modelled with a zero-inflated Beta regression. Results were reported as odds ratios with 95% confidence intervals (CIs).

**Results:**

Two hundred and thirty-two patients were included in the study. Ninety-one patients were women, 141 were men, and the mean age was 55.42 years. Neuropalliative patients represented diagnoses such as amyotrophic lateral sclerosis (ALS) (n = 81), ischemic stroke (n = 15), intracerebral haemorrhage (n = 15), Duchenne muscular dystrophy (n = 12), or craniocerebral trauma (n = 10). Palliative care counselling was the most common intervention for patients (n = 203), their close relatives (n = 177), and their nursing services (n = 75). Respiratory therapy (n = 188), speech and language therapy (n = 145), and physiotherapy (n = 143) were also frequently applied interventions. Sixty patients received botulinum toxin A treatment for hypersalivation, and 32 for spasticity. The odds of needing invasive ventilation increased by 3.7 (CI 1.7–7.8), and the need for mechanical insufflation-exsufflation increased by 2.2 (CI 1.1–4.3) in patients previously discharged from early neurological-neurosurgical rehabilitation. Prior intensive care treatment increased the odds of invasive ventilation by 5.1 (CI 2.2–11.5) and the use of mechanical insufflation-exsufflation by 2.3 (CI 1.1–4.8).

**Conclusion:**

Neuropalliative outpatient clinics demand a wide range of diagnostic measures and interventions as well as a multidisciplinary approach. Further research is necessary to investigate the relation between diagnosis and treatment needs.

**Clinical trial registration:**

https://drks.de/search/en/trial/DRKS00030778, identifier DRKS00030778.

## Introduction

### The holistic concept of palliative care

According to the definition provided by the World Health Organisation (WHO), palliative care is a clinical approach aimed at improving the quality of life for individuals with life-threatening illnesses and their close relatives (1). This includes not only relieving physical discomfort but also addressing psychological, social, and spiritual needs (1, 2). In palliative medicine, physicians alleviate the patients’ physical symptoms, for example, by administering pain medication (3). In addition, technological communication options such as telemedicine complement clinical care, which may facilitate access to consultations, especially for patients in rural areas (4).

The above-stated definition of palliative care by the WHO identifies life-threatening but non-life-limiting illnesses as conditions requiring palliative care (1). An example of a life-threatening, non-life-limiting disease is a brain stem infarction with dysphagia and the use of a tracheal cannula. When this infarction becomes chronic, it is also classified as a long-term neurological condition (LTNC). LTNCs are conditions caused by an injury or illness of the nervous system and have an impact on a person for the whole life (5).

### Indications for neuropalliative care

Demographic processes, amongst other reasons, lead to a growing number of patients with life-threatening LTNCs (3, 6). Therefore, palliative care is becoming increasingly important within neurology (7, 8). Neuropalliative care provides all interventions necessary to prevent or relieve suffering, sustain quality of life, and foster participation in patients with life-threatening neurological diseases. It also takes into account the concerns of the patient’s close relatives. Besides, neuropalliative care is a highly specialised and non-discriminating discipline (3, 9).

LTNCs treated in palliative care settings include motor neuron diseases, muscular dystrophies, dementia, brain tumours, Parkinson’s disease, multiple sclerosis (10–12), and many more. These diseases may be progressive and often have a protracted and sometimes fluctuating course (13). Moreover, acute neurological disorders such as strokes can cause life-long sequelae, for example, impaired consciousness, aspiration, spasticity, or pain (14–17). In addition, it is not easy to identify a separate dying phase in patients with LTNCs (18). Therefore, patients with neurological diseases may require palliative care treatment for a longer time than patients with other life-threatening illnesses (19). As the disease progresses, patients have needs and complications that differ from those of other diseases, such as terminal cancer (2, 7, 10). Furthermore, patients whose diseases are not neurological also show neurological and/or neuropsychiatric symptoms in the palliative stage (20). These symptoms include, for example, sensory complaints (“tightness, tingling, burning”) [(20), p. 4] or sleep disorders.

### Palliative care interventions in neurological patients

Due to the complexity of patients’ needs, treatment from different disciplines is required (3, 12). Therefore, strictly separating medical concepts of neurology, rehabilitation, and palliative care is inappropriate for patients with LTNCs (5, 21). A systematic review of 68 studies (11) showed that a multidisciplinary treatment approach in palliative care facilities reduces disease symptoms and improves the quality of life for people with chronic and progressive neurological diseases. The guideline on palliative care for neurological diseases published by the German Neurological Society therefore states that “multidisciplinary teams of modern palliative care perceive themselves as teams for a better quality of life” [(22), p. 13]. Palliative care teams may include nursing, occupational therapy, physiotherapy, psychotherapy, speech and language therapy, respiratory therapy, augmentative and alternative communication (AAC), counselling for relatives, and medical treatment.

In occupational therapy, activities of daily living (ADL), such as personal hygiene, are adapted and supported so patients can perform them independently for as long as possible (12, 23). Moreover, equipping patients with supportive devices and teaching them how to use these devices enables them to live more comfortably at home (23). Physiotherapists provide movement exercises, transfer training, and manual therapy to maintain muscle function, address comorbidities, and reduce pain (24, 25). Psychotherapy helps patients and their relatives to develop coping strategies for depression, anxiety, or cognitive impairments (3, 5). Speech and language therapists assess the patients’ swallowing ability and manage tracheal cannulas if required (26). Dysphagia and the inability to clear saliva from the respiratory system can lead to sialorrhea (27). Sialorrhea can be treated with dysphagia therapy, anticholinergic medication, or an injection of incobotulinumtoxin A into the salivary glands (27, 28). Botulinum neurotoxin A is injected by physicians into muscles affected by spasticity (29). Respiratory therapists treat and monitor ventilatory pump failure, cough insufficiency, tracheobronchial retention of secretions, and dyspnea (30). They evaluate the need for mechanical ventilation and apply the device in accordance with the patients and physicians (30). The use of mechanical insufflation-exsufflation (MI-E) is established for neuromuscular patients with impaired cough (31). In patients with neuromuscular diseases such as amyotrophic lateral sclerosis (ALS), the ability to communicate verbally deteriorates with the progress of the disease (32, 33). Therefore, in the early stages of the disease, it is important to ascertain the patients’ wishes while they can still communicate verbally. When equipped with AAC devices, patients can also interact via touch screens or eye tracking (34). As neurological diseases affect social networks, counselling of caregivers and family members plays a crucial role in palliative care (35–37). Physicians provide information about the disease and prognosis and conduct physical examinations (38). They also treat concomitant symptoms with medication and adjust the medication to the patient’s condition (3, 39).

### Outpatient neuropalliative care

The appropriate way to provide palliative care is currently a subject of discussion. De Boer et al. (40) showed that relatives find palliative care more bearable when it takes place in a hospice or at home compared to a hospital or residential care for the elderly. Kluger et al. (41, 42) conducted a randomised controlled study with 210 patients with Parkinson’s disease and 175 caregivers. One group received treatment from a neurologist primary care practitioner, while the other group received outpatient palliative care treatment with a palliative neurologist, a palliative medicine physician, a nurse, a social worker, and a chaplain. Six months after the study onset, the group receiving palliative care treatment showed a significantly higher quality of life, a significantly reduced burden of non-motor symptoms, and a significantly reduced severity of motor symptoms. The caregiver burden showed no significant differences between the groups after 6 months. However, after 12 months, caregiver burden and anxiety were significantly lower in the group with palliative care treatment. These findings emphasise the role of outpatient palliative care clinics in supporting patients to live at home. In addition, modern technologies such as telemedicine in neuropalliative care, as previously mentioned, along with robotics and brain-computer interfaces in ALS, are current research topics in outpatient neuropalliative care (43, 44).

On July 1st 2017, a *Neuropalliative Outpatient Clinic* was opened at the Evangelical Hospital Oldenburg, providing multidisciplinary care. However, some patients spontaneously reported that they were initially hesitant to attend the clinic because they associated the term “palliative” with end-of-life care, even though they felt they were not dying yet. For example, one patient stated: “I do not want to attend a palliative care clinic because this would mean giving up.” Since the most common diagnosis at that time was ALS, a second outpatient clinic was established and called *Outpatient Clinic for Amyotrophic Lateral Sclerosis and Severe Neuromuscular Diseases*. This means that the same treatment concept was still being offered under two different clinic names. Kluger et al. (38) also reported renaming their *Neurology Palliative Care Clinic* to *Neurology Supportive and Palliative Care Clinic* due to misinterpretations of the patients and the referring clinicians regarding the term “palliative“. Avoiding palliative care due to the association with hopelessness or abandonment can have sociocultural reasons, including how society copes with death (45).

### Objectives

In 2022, the American Academy of Neurology (AAN) issued a position statement on clinical guidance in neuropalliative care, delivering perspectives on future development of neuropalliative care (46). In 2023, the “DGN Practise Guideline on Palliative Care in Neurological Disease (S2k)” (22) provided key recommendations for palliative treatment. In addition, the palliative care needs of people with intellectual and complex impairments have now been recognised by the German Society for Palliative Medicine.

To our knowledge, data on the clinical characteristics of the patient clientele in outpatient neuropalliative care clinics are limited to a publication by Kluger et al. (38), a brief overview by Steinker and Groß (47), and a letter by Thomas et al. (48). Additionally, Phillips et al. (49) reported on the implementation of outpatient neuropalliative care in 6 ALS clinics in the United States. Systematic accounts of the treatment spectrum in outpatient neuropalliative care clinics are thus needed. This study was developed to add knowledge in this regard.

This study has three aims:

1. To describe the patients with palliative care needs treated in our *Neuropalliative Outpatient Clinic* and *Outpatient Clinic for Amyotrophic Lateral Sclerosis and Severe Neuromuscular Diseases*.
2. To describe the outpatient clinic’s clinical treatment spectrum and healthcare delivery according to the patients’ diagnosis.
3. To investigate the association of patient characteristics and respiratory treatments.

## Materials and methods

### Study design

An analysis of clinical routine data from patients with palliative care needs was conducted. The patients visited the *Neuropalliative Outpatient Clinic* and the *Outpatient Clinic for Amyotrophic Lateral Sclerosis and Severe Neuromuscular Diseases* of the Evangelical Hospital Oldenburg from July 1st 2017 to June 30th 2022.

### Inclusion and exclusion criteria

Study patients were required to meet one of the following two characteristics: (1) the presence of a severe neurological disease with palliative care needs according to the WHO definition (1) of palliative care, or (2) having a life-threatening neuromuscular disease (e.g., motor neuron disease or Duchenne muscular dystrophy [DMD]). Patients younger than 18 years of age and those without palliative treatment needs were not included in the study.

### Neuropalliative care treatments

Depending on their needs, patients received interventions from a multidisciplinary and interprofessional team. The treatment of the participants was conducted in accordance with the standards of the local outpatient clinic, which applied to all patients. The outpatient clinic was established collaboratively by the multidisciplinary teams of the *Interdisciplinary Palliative Care Centre* and the *Clinic for Neurological Intensive Care and Early Rehabilitation*. Consequently, multidisciplinary care provided by experienced experts was ensured from the very beginning.

The team consisted of a neurologist certified in palliative medicine, palliative care nurses, speech and language therapists, respiratory therapists, occupational therapists, physiotherapists, and an augmented and alternative communication (AAC) expert. The occupational therapists and physiotherapists had expertise in assistive technology. Before the patients’ arrival, a team conference was held to review the available information about the patients, clarify which team members were needed for treatment, and schedule multidisciplinary work during the visit. When patients entered the outpatient clinic, they and their close relatives or accompanying nurses/assistants were welcomed by the neurologist and nurses. Then, the current health status was elaborated, and a detailed psychosocial history was taken.

Palliative care counselling comprised communicating the disease prognosis, explaining the impact of life-supporting technologies in progressive disease, and assisting with decision-making during clinically relevant changes. For example, in ALS, clinical milestones are established, some of which require the patient to decide on issues such as artificial nutrition, noninvasive ventilation (NIV), or tracheotomy (50). In our outpatient clinic, patients were also counselled on how to document their wishes, for example, by means of an advance directive. Particular attention was given to whether patients could participate in society, as it was important to them. This also included, for example, finding suitable employment. Additionally, patients were offered peer counselling or contact with other patients, for example, through connections with disability associations or self-help groups.

Overall, counselling aimed at improving patients’ health status and quality of life. In addition, the role of caregiving was discussed with close relatives. An important clinical aspect was managing impaired swallowing, coughing, and breathing. Speech and language therapists provided flexible endoscopic evaluation of swallowing (FEES) and tracheoscopy to assess patients’ swallowing ability. Mechanical ventilation, initiated in the outpatient clinic or a hospital, was controlled by inspecting the settings on the ventilation device. If necessary, the settings were modified, too. Invasive mechanical ventilation was always administered via a tracheal cannula.

To reduce salivation, incobotulinumtoxin A was injected into the patients’ salivary glands. Incobotulinumtoxin A, abobotulinumtoxin A, and onabotulinumtoxin A were used to alleviate spasticity and injected into various muscles. All injections were guided by ultrasound. Orthopaedic technicians, otorhinolaryngologists, and cardiologists were consulted if required.

### Data acquisition and processing

Information on patient treatment was entered and stored directly in the hospital’s digital information system (Orbis, Daedalus, DH Healthcare, Bonn, Germany) after the treatment appointments. Relevant information was extracted from this data for this study. This means, data of patients treated from July 1st 2017 to June 30th 2022 were retrieved. A palliative care nurse from the outpatient clinic collected all this information and entered the anonymized information into the programme *Research Electronic Data Capture* (REDCap, version 14.5.36) (51). REDCap is a browser-based software for creating and managing research databases. Only those team members involved in the project had password-protected access to REDCap. Data processing was performed on the university’s IT services systems (transport encryption TLS 1.2). After data collection and correction, the data were exported as a CSV- and R-file for statistical analysis.

### Obtained data

Clinical routine information on mechanical ventilation, the presence of a percutaneous endoscopic gastrostomy (PEG) tube, and the use of a tracheal cannula at the first appointment was collected. We also determined what treatment the patients had received before visiting our outpatient clinic, such as no treatment, early neurological-neurosurgical rehabilitation (ENNR), neurological follow-up rehabilitation, acute neurological care, treatment in an intensive care unit (ICU), or other treatments. Moreover, the following need-specific interventions were recorded:

- Palliative care counselling.
- Respiratory therapy.
- Speech and language therapy.
- Physiotherapy.
- Occupational therapy.
- Augmentative and alternative communication (AAC) / technically aided communication.
- Administration of botulinum neurotoxin type A into the muscles.
- Administration of incobotulinumtoxin A into salivary glands.
- Consultation with a cardiologist.
- Initiation of noninvasive ventilation (NIV) or control of the device settings for NIV.
- Initiation of invasive ventilation (IV) or control of the device settings for IV.
- Initiation of mechanical insufflation-exsufflation (MI-E) or control of the device settings for MI-E.
- Flexible endoscopic evaluation of swallowing (FEES).
- Tracheoscopy.

In addition, the frequency of appointments between July 1st 2017 (quarter 3 in 2017) and June 30th 2022 (quarter 2 in 2022) was obtained.

### Statistical analysis

The data were analysed using the statistical programme R (version 4.3.2, GNU General Public Licence, Free Software Foundation). Patient characteristics and the range of treatments were described and grouped by the disease’s location of the lesion. Categorical variables were reported as absolute and relative frequencies. The median and interquartile range (IQR) were calculated for metric variables.

We also examined the effort of the following treatments: IV, NIV, and MI-E. For this purpose, the total number of treatments was divided by the number of quarters in which a patient received treatment at the outpatient clinic. This was conducted both for the initiation of the respective treatment (with IV, NIV, or MI-E) and for the control of the device settings. No effort was defined as no treatment performed during the entire time of care; minimal effort was defined as a single overall treatment of that kind; and high effort was defined as at least one treatment performed per quarter. We modelled these variables with a mixed distribution.

After inspection of the distributions of the data, we chose a zero-inflated Beta distribution. To analyse this distribution, we constructed separate generalised additive regression models for location, scale, and shape (GAMLSS) for each effort variable. These models contained one predictor for the probability that no treatment of this type was required compared to one treatment of that kind. Also, the models contained another predictor for the probability of high effort of treatment with at least one treatment per quarter compared to a single treatment overall. We pre-selected a set of potential covariates for each model based on medical importance. Automatic model selection was then performed in a bidirectional stepwise procedure based on Akaike’s Information Criterion (AIC). The resulting final regression coefficients were presented as odds ratios (ORs) with 95% confidence intervals (CIs).

### Ethics and data privacy

The study was approved by the ethics committee of the Carl von Ossietzky Universität Oldenburg (identifier 2022-165) and conducted in accordance with the *Declaration of Helsinki* (52). Moreover, it was registered in the *German Clinical Trials Register* (clinical trial identifier DRKS00030778). Health data were processed anonymously. No informed consent was required to be obtained, as processing of clinical patient data for research purposes without informed consent is justified by § 7 IX DSG-EKD, § 13 II Nr. 10 DSG-EKD, and § 17 IV DSG-EKD, provided that subsequent collection of informed consent would be unreasonably complicated or impossible. The anonymised data were stored on the hospital’s and the university’s computers. Access to the data was restricted to the project team members. The data will be deleted on the 1st of June 2032 at the latest.

## Results

### Participants

**Figure 1:**
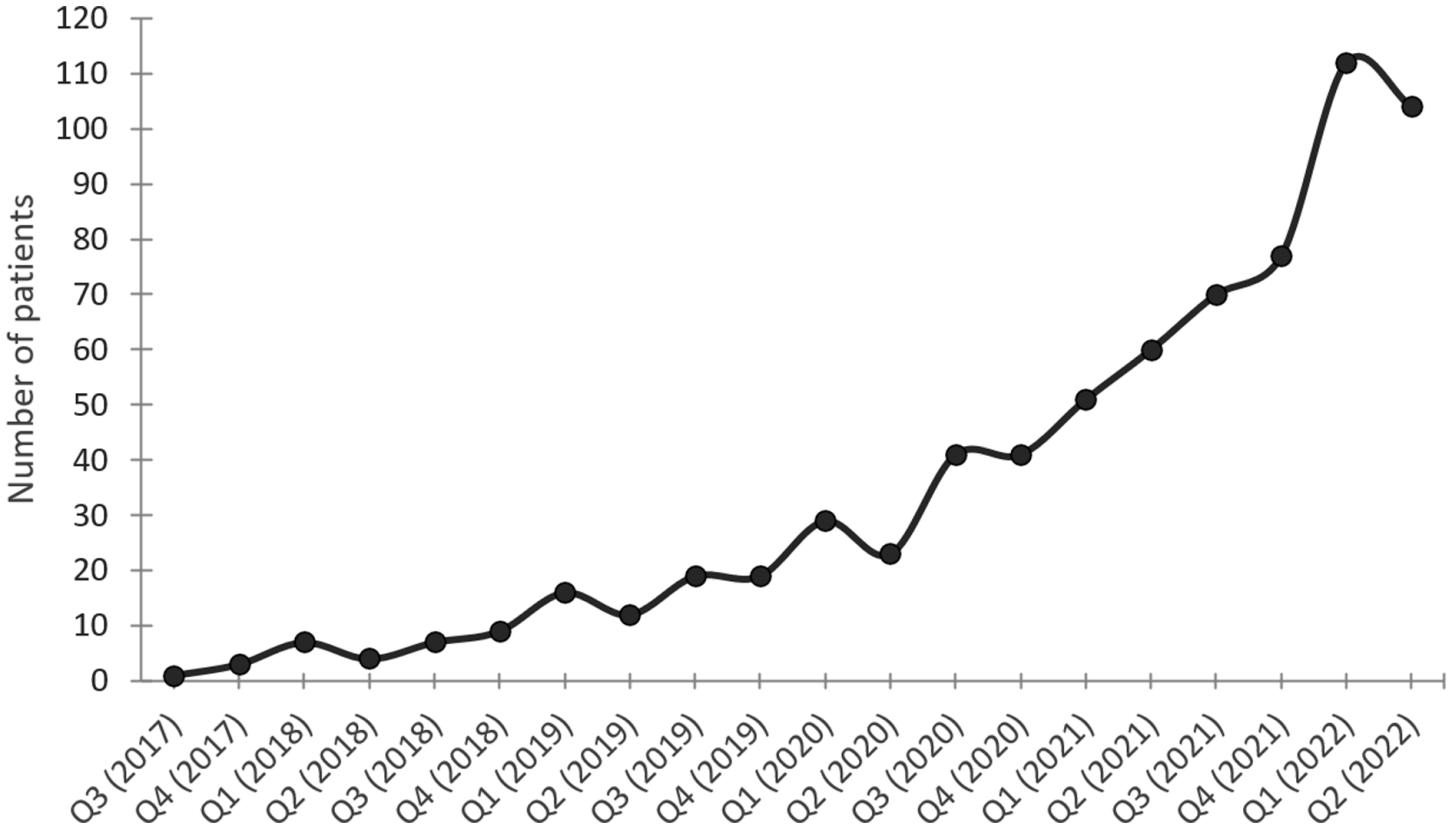
Number of patients treated per quarter during the study phase.

Since the opening of the outpatient clinic on July 1st 2017 until June 30th 2022, the number of patients fulfilling the inclusion criteria for the study has steadily increased (see Figure 1). Two hundred and thirty-two patients who were treated in our clinic were included in the study.

The age of the participants ranged between 18 and 89 years (mean [M] = 55.42; standard deviation [SD] = 19.82). Detailed characteristics of the participants are grouped by the disease’s location of the lesion in Table 1. Due to new findings, the non-motor neuraxis is impaired in ALS, and a cortical involvement is discussed, wherefore it cannot be defined exclusively as a neuromuscular disease (53, 54). For analytical reasons, ALS is categorised as a neuromuscular disease in Table 1 and further statistical calculations.

**Table 1:** Description of the participants.

Variable	Values	Brain lesion	Spinal lesion	Neuromuscular	Total
Gender	Female	26	9	56	91
	Male	52	11	78	141
Total	Sum female and male	78	20	134	232
Age	Median (IQR^a^)	60 (46–71)	41 (28–66)	61 (48–70)	60 (44–70)
Smoking	Never	33	16	76	125
	Previously	37	2	41	80
	Current	8	2	17	27
Family	Parship	41	10	87	138
	Single	35	10	46	91
	Unknown	2	0	1	3
Disease origin	Genetic	1	6	57	64
	Autoimmune	2	1	0	3
Onset	Birth	7	1	3	11
	Childhood	5	6	30	41
	Adult	66	13	101	180
Charlson Comorbidity Index	Median (IQR)	6 (4–9)	3 (1–6)	3 (2–5)	4 (2–7)

a IQR: interquartile range.

**Figure 2:**
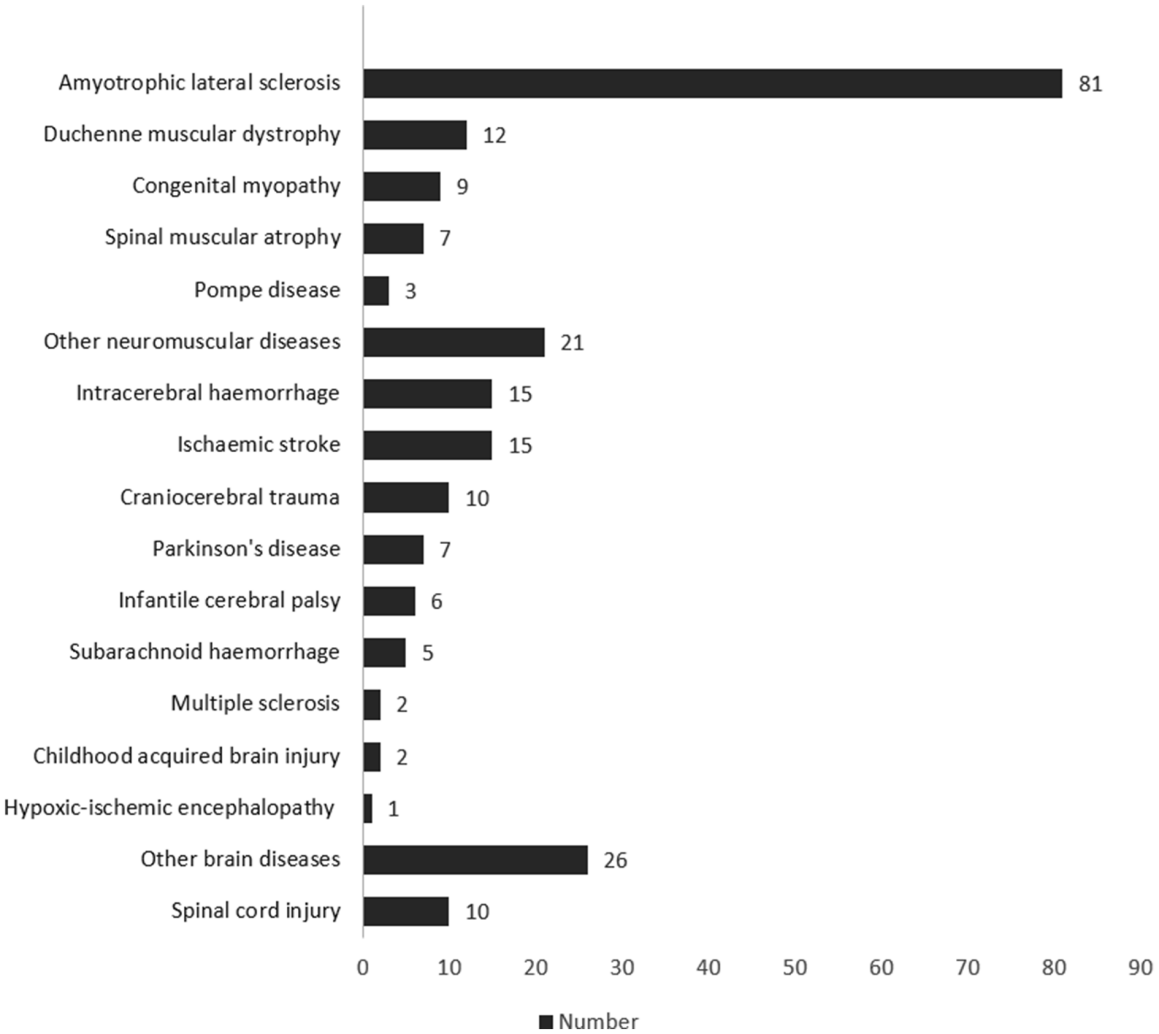
Specified patient diagnoses (number).

Figure 2 shows all diagnoses of the patients. A genetic disorder was present in 64 cases (27.59%), and an autoimmune disease in 3 cases (1.29%). Other diseases were metachromatic leukodystrophy, Huntington’s disease, schizencephaly, or encephalitis. The most common comorbidities as reported in the Charlson Comorbidity Index (55) were systolic heart insufficiencies (n = 104; 44.83%), peripheral vascular diseases (n = 93; 40.09%), and cerebrovascular diseases (n = 61; 26.29%). Fifty-nine (25.43%) of the patients had a chronic lung disease such as obstructive pulmonary disease (COPD) or asthma as a concomitant illness. The mean of the Charlson Comorbidity Index of all participants was 4.64 (SD 3.41), and the median was 4 (IQR: 2–7).

Most participants lived at home supported by close relatives (n = 108; 46.55%). Other patients lived in an intensive care shared apartment (n = 45; 19.40%) or at home with intensive care (n = 38; 16.38%). One hundred and thirty-eight (59.48%) patients were in a partnership, 91 (39.22%) were single, and in 3 (1.29%) cases, the family status was not documented. Forty-one of the participants (17.67%) lived in Oldenburg, 18 (7.76%) in Bremen, and 5 (2.16%) in the area around Oldenburg. Most participants (n = 168; 72.41%) came from other regions, mainly in Lower Saxony, with a median distance of 53 (IQR 33–76) road kilometres (33 miles) from their place of residence to our outpatient clinic. Eleven (4.74%) patients had their disease since birth, 41 (17.67%) since childhood, and in 180 (77.59%) patients, the disease developed in adulthood. Twenty-seven (11.64%) of the patients were smokers, 80 (34.48%) smoked in the past, and 125 (53.88%) never smoked. When the patients first visited the outpatient clinic, 26 (11.21%) participants already received noninvasive ventilation, and 37 (15.95%) received invasive ventilation. Thirty-five (15.09%) patients had a tracheal cannula without invasive ventilation, and 134 (57.76%) neither needed a tracheal cannula nor mechanical ventilation. Seventy-eight (33.62%) of the patients were receiving nutrition via percutaneous endoscopic gastrostomy (PEG). Fifty-four (23.28%) of the patients died during the survey phase, and 178 (76.72%) were alive at the end of the survey phase.

### Frequency of treatments

Two hundred and three (87.50%) patients, 177 (76.29%) close relatives, 75 (32.22%) accompanying nurses/assistants, and 11 (4.74%) other persons received detailed palliative care counselling. Of the patients, 143 (61.64%) were seen by a physiotherapist, 71 (30.60%) were seen by an occupational therapist and 28 (12.07%) were supported with AAC during the course of the treatment in the neuropalliative outpatient clinic (see Table 2). Respiratory therapists were consulted for 188 (81.03%) of the patients. Noninvasive mechanical ventilation was initiated in 20 (8.62%) and invasive mechanical ventilation in 11 (4.74%) patients. Table 3 shows age, gender and the presence of mechanical ventilation amongst different diagnoses. Fifty (21.55%) patients received an implementation of a mechanical insufflation-exsufflation (MI-E).

**Table 2:** Diagnostics and treatments.

Variable	Number of participants (frequency in %)
Palliative care counselling for patient	203 (87.50%)
Palliative care counselling for relatives/friends	177 (76.29%)
Palliative care counselling for nursing service	75 (32.22%)
Palliative care counselling for other person	11 (4.74%)
Respiratory therapy	188 (81.03%)
IV^a^ initiation	11 (4.74%)
NIV^b^ initiation	20 (8.62%)
MI-E^c^ initiation	50 (21.55%)
Speech and language therapy	145 (62.50%)
Physiotherapy	143 (61.64%)
Occupational therapy	71 (30.60%)
Cardiologist consultation	24 (10.34%)
Augmentative and alternative communication	28 (12.07%)
PEG^d^ change	9 (3.88%)
Injection of incobotulinumtoxin A into salivary glands	60 (25.86%)
Injection of botulinum neurotoxin A into muscles	32 (13.79%)
Tracheoscopy	72 (31.03%)
FEES^e^	104 (44.83%)

a IV: invasive ventilation.

b NIV: noninvasive ventilation.

c MI-E: mechanical insufflation-exsufflation.

d PEG: percutaneous endoscopic gastrostomy.

e FEES: flexible endoscopic evaluation of swallowing.

Number of patients with the respective diagnostics/treatment during the survey phase.

**Table 3:** Age, gender, and presence of mechanical ventilation amongst different diagnoses.

Underlying condition	Median age (IQR)^a^	Gender		Pre-treatment		Initiation	
		f^b^	m^c^	IV^d^	NIV^e^	IV	NIV
ALS^f^	65 (57–72)	31	50	9	12	6	15
DMD^g^	21 (19–23)	0	12	4	6	1	1
Congenital myopathy	20 (19–26)	7	2	1	3	1	2
Spinal muscular atrophy	36 (19–50)	3	4	0	1	0	0
Pompe disease	62 (59–65)	2	1	0	1	0	1
Other neuromuscular diseases	55 (27–72)	11	10	4	0	2	1
SCI^h^	65 (31–74)	4	6	6	2	0	0
All brain diseases	60 (44–71)	33	56	13	1	1	0
Total	60 (44–70)	91	141	37	26	11	20

a IQR: interquartile range.

b f: female.

c m: male.

d IV: invasive ventilation.

e NIV: noninvasive ventilation.

f ALS: amyotrophic lateral sclerosis.

g DMD: Duchenne muscular dystrophy.

h SCI: spinal cord injury.

In addition, 145 (62.50%) patients were seen by a speech and language therapist. A FEES was conducted for 104 (44.83%) of the patients. Seventy-two (31.03%) patients received a tracheoscopy. A PEG tube was present in 96 (41.38%) of the patients. In 9 (3.88%) of the patients, the PEG tube was changed, and in 5 (2.16%) of the patients, it was removed. Incobotulinumtoxin A was injected into the salivary glands in 60 (25.86%) patients. Twenty of the patients who had received an injection of incobotulinumtoxin A into the salivary glands had ALS. Incobotulinumtoxin A, abobotulinumtoxin A, and onabotulinumtoxin A were injected into various muscles of 32 (13.79%) patients. Finally, the cardiologist was consulted for 24 (10.34%) of all patients. Four of 12 patients with Duchenne muscular dystrophy received a cardiology consultation.

### Association of patient characteristics and respiratory treatments

The results of the GAMLSS regression for the treatment effort are reported in Table 4. The results contain odds ratios (ORs) and confidence intervals for three separate models differing by their response variable: effort for the initiation and control of IV, NIV, and MI-E as defined in the methods section. In addition, the results comprise the OR for high treatment effort compared to minimal treatment effort, as well as the OR for at least minimal treatment compared to no treatment at all. In the joint model selection, age, gender, and the disease’s location of the lesion were selected as predictors for high treatment effort. Gender, prior treatment (as described in the chapter *Obtained Data*), and location of lesion were selected as predictors for the necessity of at least one treatment (i.e., presence of treatment effort) compared to zero necessary treatments.

**Table 4:** Effort for initiation or control of invasive ventilation, noninvasive ventilation, and mechanical insufflation-exsufflation.

Variable	Values	Invasive ventilation	Noninvasive ventilation	MI-E^a^
Model part	High effort vs. minimal effort			
Age	In years	1.02 (1.00–1.03)	1.01 (0.99–1.02)	1.01 (0.99–1.02)
Gender	Female	0.65 (0.34–1.24)	1.37 (0.62–3.03)	0.82 (0.50–1.33)
Location of lesion	Brain	1.27 (0.64–2.53)	2.22 (0.59–8.29)	1.24 (0.71–2.17)
Model part	Minimal effort vs. no effort			
Gender	Female	0.60 (0.29–1.23)	1.86 (0.88–3.96)	0.93 (0.52–1.66)
Prior treatment	ENNR^b^	3.69 (1.74–7.81)	0.94 (0.37–2.37)	2.18 (1.11–4.30)
	ICU^c^	5.06 (2.22–11.50)	1.20 (0.46–3.18)	2.32 (1.12–4.81)
Location of lesion	Brain	0.32 (0.14–0.73)	0.09 (0.03–0.32)	0.24 (0.12–0.48)

a MI-E: mechanical insufflation-exsufflation.

b ENNR: early neurological-neurosurgical rehabilitation.

c ICU: intensive care unit.

No effort: no treatment performed during the whole time of care; minimal effort: a single overall treatment of that kind; high effort: at least one treatment performed per quarter. Results of regression (OR with 95% CI).

The regression coefficients showed that patients with previous early neurological-neurosurgical rehabilitation (ENNR) treatment had a 3.69 times increased probability of needing IV and an OR of 2.18 for requiring MI-E. Also, prior ICU treatment significantly increased the odds of needing IV (OR 5.06) and MI-E (OR 2.32). Patients with brain lesions were significantly less likely to require IV (OR 0.32), NIV (OR 0.09), or MI-E (OR 0.24) compared to patients with neuromuscular diseases or spinal lesions.

## Discussion

### Neurological Patients with Palliative Care Needs Cover a Wide Range of Progressive and Non-Progressive Diseases and Are Dependent on Regional Healthcare Structures

The results indicate that most participants were male (61%), and the average age was 55 years. Very few participants had their disease from birth, with most experiencing disease onset in adulthood. A variety of different congenital and acquired neurological conditions were treated, with the most frequent disease being ALS, accounting for about one-third of the patients. This differs from the distribution of neurological diseases in Europe, where the most common neurological diseases are stroke and dementia (56). With a prevalence of about 8 per 100,000 person-years, ALS is considered a rare disease (57). Nonetheless, many patients with ALS were referred to our neuropalliative outpatient clinic due to progressive difficulties with swallowing, cough, respiration, and other related symptoms. This was likely because our outpatient clinic was the only institution in the vicinity of the cities of Oldenburg and Bremen specializing in the treatment of ALS and severe neuromuscular diseases. In addition, patients with ischaemic stroke, intracranial haemorrhage, and DMD were amongst the most frequently treated. DMD is a disease that occurs almost exclusively in men (58), and ALS affects males more often than females (57, 59).

A stroke occurs suddenly and can lead to severe disabilities, with palliative treatment needs differing from those of diseases with a gradual onset (60). Palliative care may be appropriate shortly after the stroke or later in the chronic phase if severe complications develop. Steigleder et al. (60) recommend standardised screenings for palliative care needs at regular intervals for stroke patients. Therefore, neuropalliative care is necessary for both patients with progressive neurological conditions and those with acute neurological diseases.

Neuropalliative care must also consider comorbidity. In the present study, the median Charlson Comorbidity Index was 4, which has been associated with a one-year mortality rate of 52% in the initial validation study (55). Comorbidities further worsen the prognosis of the underlying neurological disease, as demonstrated for heart failure in ALS patients and COPD in stroke patients (61, 62). Additionally, comorbidities may exacerbate existing symptoms or cause new symptoms that require palliative treatment.

Forty-one of the patients came from Oldenburg, and most patients came from other towns in Lower Saxony to our outpatient clinic. Therefore, patients as well as family caregivers, nurses, and other health professionals accompanying them to the visits, were willing to travel considerable distances to reach the clinic. Neuropalliative care should be established regionally to reduce the burden and risks associated with patient transport (63, 64), with the latter being best understood in the context of interhospital transport. Furthermore, establishing regional multidisciplinary outpatient clinics might prevent frequent admissions to acute care hospitals.

### Palliative care counselling and symptom-oriented therapies

Palliative care counselling for patients by the palliative care nurse or the neurologist was the most frequent intervention. Palliative care counselling should take into account that “the effects of disease in any part of the individual’s being will impact on all other areas of the self” [(65), p. 9]. This can be achieved, for instance, by providing adequate support in every phase of the disease and involving caregivers (3). A compassionate view of the condition helps patients feel validated and understood (66). Patients also benefit from honest and clear communication with reassuring questions, leading from compassion to action (67, 68). Trustworthy and appreciative physician-patient and nurse–patient relationships form the foundation for patient-centred treatment (69, 70).

Communication, following the above-mentioned principles, is also essential in decision-making. In our outpatient clinic, decision-making was a considerable part of palliative care counselling. Decision-making in a palliative context encompasses choices about care, medical procedures and end-of-life decisions (71). Additionally, early AAC counselling is beneficial. AAC devices can support individuals who cannot communicate verbally, enabling them to express and modify their wishes as circumstances change (72). In our study, 28 patients received counselling regarding existing or upcoming AAC device usage.

Some of our patients reached a condition where they no longer wished to be supported by life-sustaining technologies. This refusal can be expressed through an advance directive. Legally authorised persons, such as family members, can also communicate the patient’s presumed will. The opinions of family members are particularly important when the patient is cognitively impaired and unable to give informed consent. This important and personal decision must be carried out in accordance with legal requirements. For example, legal regulations in Germany permit the non-implementation or discontinuation of life-supporting technologies if the patient, who is capable of making informed decisions, rejects these interventions (73).

When patients participate in decisions regarding their care, they are more content with their treatment (71). Moreover, decision-making and patient participation contribute to patient empowerment (74). Consequently, decision-making in neuropalliative care necessitates a patient-centred and family-oriented approach.

In patients with disorders of consciousness, carefully watching non-verbal signals helps to evaluate the patients’ overall health and well-being. Such small signals can include changes in breathing, opening or closing eyes, sweating, and signs of tension or relaxation (75). Close relatives can become skilled at recognising these subtle signals and should be encouraged to share their observations with the clinical staff. When observing patients’ signals and addressing their families’ needs, nurses also have a crucial function (18).

The second most common therapy for our patients was treatment by a respiratory therapist. Respiratory therapists are involved in initiating and controlling NIV, IV, and MI-E. NIV reduces symptoms, enhances quality of life, and extends life in patients with neuromuscular diseases and subsequent ventilatory pump failure (76). Indications for the initiation of NIV in ALS and other neuromuscular diseases have been published in various guidelines (77, 78). In patients with ALS and severe bulbar impairment, NIV does not prolong life, but it improves quality of life, especially with regard to sleep-specific symptoms (79). Manually assisted coughing, air stacking, and MI-E can aid in managing secretions in impaired cough, thereby reducing dyspnoea and preventing pneumonia (76, 80, 81). MI-E often proves ineffective in patients with bulbar ALS due to laryngeal adduction; therefore, customised MI-E settings are necessary (82). Tracheotomy is indicated when ALS patients develop hypoxic respiratory failure despite treatment with NIV and MI-E (76, 83). In Germany, the quality of life for patients with ALS undergoing NIV or IV via a tracheal cannula is generally good. However, IV places more burden on caregivers (84).

Treatment from speech and language therapists and physiotherapists was needed by more than 60% of the patients. The need for speech and language therapy was mainly due to the necessity for specific endoscopic diagnostics, such as FEES and/or tracheoscopy (see Table 2). Speech and language therapists also provided advice to the patients on nutrition and the management of tracheal cannulas, respectively. Occupational therapy was needed by about 30% of the patients. Surveys involving professionals and patients indicate that the therapeutic offers of physiotherapists and occupational therapists meet many of the patients’ needs, yet there are currently too few of these therapists working in palliative care (85–87). Although the qualifications and specialisations of these two professional groups differ, their tasks often overlap (87). For example, in our outpatient department, both professions provided the adaptation of assistive devices and therapy of spasticity in the outpatient setting. The variety of speech and language therapy, physiotherapy, and occupational therapy interventions for the patients in our study shows the need for multidisciplinary therapy in neuropalliative patients. Brennan et al. (88) suggest that neurodegenerative diseases may be treated by a multidisciplinary team, as a wide range of interventions is necessary to provide the most suitable treatment for each disease. In addition, the European Academy of Neurology (EAN) guideline on the management of ALS outlines the healthcare professionals needed in a multidisciplinary team to address ALS symptoms (78).

### Factors influencing the need for mechanical ventilation or mechanical insufflation-exsufflation

Patients with neuromuscular diseases or spinal lesions were significantly more likely to receive treatments with IV, NIV, or MI-E compared to patients with a brain disease (see Table 4). This variation in the use of mechanical ventilation or MI-E is probably due to the absence of ventilatory pump failure in brain disease. However, acquired hypoventilation syndrome (AHS) or disorders of respiratory rhythm may occur in brainstem disease (89, 90). AHS often requires mechanical ventilation, and it is a rare syndrome (91).

There was also a significantly higher probability of receiving initiation or control of IV or MI-E in patients previously treated in ICUs or ENNR (see Table 4). Treatment in an ICU may imply high overall disease severity, while treatment in ENNR indicates high overall disease severity combined with delayed recovery (92). Therefore, it can be emphasised that neurological patients after treatment in ICUs and ENNR may require neuropalliative care.

Age did not affect the need for mechanical ventilation or MI-E (see Table 4). Amongst our patients, two groups were particularly likely to require mechanical ventilation: ALS, which usually occurs in adults, and DMD, which begins in childhood. Specifically, ALS is a disease of the elderly, with a mean onset age of 67 years (57). Most ALS patients in our study did not initially need mechanical ventilation at the outpatient clinic (see Table 3). However, over the course of treatment, the number of ALS patients needing mechanical ventilation increased, with most of them receiving NIV. NIV extends the lifespan of ALS patients, especially with longer daily use (93). The number of ventilated DMD patients rises with age, and by 35 years and older, about 90% are on mechanical ventilation (94, 95). Consequently, the age range of our mechanically ventilated patients was broad (see Table 3).

### Comparison with data from other neuropalliative care outpatient clinics

Kluger et al. (38) conducted a retrospective study in which diagnoses and treatments in their neuropalliative outpatient clinic were documented and analysed. A neurologist, a physician assistant, a nurse, a chaplain, and a social worker treated the patients. If necessary, the patients were referred to a psychotherapist, an acupuncturist, or other experts. In their clinic, 45 female and 51 male patients with an average age of 65 years were treated. Most of them had Parkinson’s disease (n = 34), Parkinson’s disease dementia (n = 16), and multiple sclerosis (n = 8). Other diseases were, for example, progressive supranuclear palsy, Lewy body dementia, or Alzheimer’s disease. The age and diagnoses of the patients in our setting differed considerably from those reported by Kluger et al. (38). The mean age in our study was lower due to a different spectrum of diagnoses. As Thomas et al. (48) mainly treated ALS patients, their patient clientele is more comparable to ours. ALS has an age of onset similar to that of Parkinson’s disease, but a much faster clinical progression (96, 97). Additionally, patients with severe genetic neurological conditions and early-onset life-threatening symptoms were regularly treated in our outpatient department. Kluger et al. (38) also reported that the number of patients in their outpatient clinic increased over time, as in our outpatient clinic.

Moreover, the members of the neuropalliative care team and the interventions offered varied. This may be due to differences between the studies regarding patient diagnoses and symptoms, treatment approaches, regional healthcare services, and country-specific aspects of healthcare professional education (38, 48).

### Limitations

One limitation is the monocentric design of the study. Other outpatient clinics may treat different diseases, provide different treatments, and be part of different regional healthcare systems. Another limitation is the lack of data on medication use, such as opiates or benzodiazepines. Additionally, there are no assessments of the outcomes of the interventions, including clinical results, patient-reported outcomes, and caregiver-reported outcomes.

Some comorbidities might have been overlooked as the patients came into the outpatient clinic primarily due to a neurologic disease. It is possible that some patients did not bring the medical reports of all the other physicians with them.

Additionally, this study presents a treatment approach that requires considerable financial and technical resources, as well as trained personnel. Therefore, its implementation largely depends on regional prerequisites and should be adapted accordingly. For example, when technical devices are unavailable, multidisciplinary care can still be provided.

## Conclusion

Palliative care in neurology is needed promptly after diagnosis (18, 19). Life-threatening symptoms of neurological diseases should be detected and treated early. The variety of neurological conditions that may require neuropalliative care is broad and comprises both progressive and non-progressive diseases. Furthermore, the needs of patients requiring permanent life-sustaining interventions must be addressed by neuropalliative care. Additionally, many patients have non-neurological comorbidities that also require treatment.

Neuropalliative care should encompass the entire spectrum of the patient’s needs. To achieve this, neuropalliative treatment must be provided by a multidisciplinary and interprofessional team. Palliative care counselling, specific diagnostics, and needs-oriented therapies are required. Injection of botulinum neurotoxin A for treating spasticity and hypersalivation is also part of neuropalliative care treatment. Neuropalliative care also includes the provision of FEES and tracheoscopy, as well as management of NIV, IV, and MI-E. AAC and other assistive technologies are helping patients interact with others (72). Specific expertise must be high in all members of the neuropalliative care team. This is especially important in the outpatient setting, as there is limited time to solve the patients’ issues. A comprehensive treatment with palliative care may help extend the life expectancy of patients with life-threatening acute neurological diseases or LTNCs.

Patients with life-threatening diseases and their families prefer palliative care at home (40). Therefore, opening more neurological palliative outpatient clinics could help enhance the quality of life for patients and their close relatives. Finally, more research about neurological palliative care is required to better understand the needs of the patients and their relatives and to utilise the findings in the training of healthcare professionals (98).

## Data Availability

The raw data supporting the conclusions of this article will be made available by the authors, without undue reservation.

## Glossary

AAC: augmentative and alternative communication
ADL: activities of daily living
AHS: acquired hypoventilation syndrome
AIC: Akaike Information Criterion
ALS: amyotrophic lateral sclerosis
CI: confidence interval
COPD: chronic obstructive pulmonary disease
DMD: Duchenne muscular dystrophy
EAN: European Academy of Neurology
ENNR: early neurological-neurosurgical rehabilitation
FEES: flexible endoscopic evaluation of swallowing
GAMLSS: generalised additive model for location, scale, and shape
ICU: intensive care unit
IQR: interquartile range
IV: invasive ventilation
LTNC: long-term neurological condition
M: mean
MI-E: mechanical insufflation-exsufflation
NIV: noninvasive ventilation
OR: odds ratio
PEG: percutaneous endoscopic gastrostomy
REDCap: Research Electronic Data Capture
SCI: spinal cord injury
SD: standard deviation
WHO: World Health Organisation
